# Re-examining hypotheses concerning the use and knowledge of medicinal plants: a study in the Caatinga vegetation of NE Brazil

**DOI:** 10.1186/1746-4269-2-30

**Published:** 2006-07-26

**Authors:** Ulysses Paulino de Albuquerque

**Affiliations:** 1 Laboratório de Etnobotânica Aplicada, Área de Botânica, Departamento de Biologia, Universidade Federal Rural de Pernambuco, Rua Dom Manoel de Medeiros s/n, Dois Irmãos – Recife – Pernambuco –, 52171-030, Brasil

## Abstract

**Background:**

The Caatinga (dry land vegetation) is one of the most characteristic vegetation types in northeastern Brazil. It occupies a large percentage of the semi-arid region there, and generally supports two major types of economic activity: seasonal agriculture and the harvesting of plant products. However, very little information is available concerning the interaction of people with the plants of the Caatinga.

**Methods:**

A study was undertaken with the participation of 31 adults from a rural community in the state of Pernambuco, Brazil, in order to analyze the patterns of use of medicinal plant resources, and to test a number of hypotheses concerning their use and local knowledge about them. The sources of medicinal plants used by the local community, the differences in oral information concerning the use of plants with their effective uses, and the role of exotic plants in local folk medicine practices were examined.

**Results:**

Forty-eight plant species were cited as having medicinal uses, of which 56.25% are native to the Caatinga region. The patterns of harvesting and the importance of these trees and shrubs as medicinal plants seem to be compatible with a hypothesis based on the seasonal availability of plant resources. There is no direct correlation between known medicinal plants and those used by the local population, which agrees with observations made in different tropical regions. However, this observation was not interpreted in terms of the idea of "erosion" of knowledge (commonly used to explain this lack of correlation), but rather to propose two new concepts: "mass knowledge" and "stock knowledge".

**Conclusion:**

Native plants are a very significant component of locally used medicinal plants, although exotic plants are important for treating specific health problems – which leads the proposal of a hypothesis of diversification.

## Background

Patterns of medicinal plant use by local peoples are considered to vary as a function of plant habitat collection, cultural changes, and ecological and biochemical aspects [1,2]. For example, on one hand the literature suggests that the most probable explanation for the dominance of weeds in different medicinal floras is that they have high levels of bioactive compounds. This idea is based on the apparency hypothesis [see [3-5]]. The central idea is the following: "the plants could be classified into two basic groups: 'apparent', and 'non-apparent'. 'Apparent' plants are usually perennial woody plants or those that are normally dominant in the ecosystem; while the 'non-apparent' plants would be represented by the herbaceous species (principally small plants) with a short life cycle, as well as those present in the early stages of ecological succession. It is presumed that 'apparent' plants will invest in low-molecular weight compounds that are highly bioactive (qualitative defense), such as alkaloids, which would be produced in relatively low concentrations" [5]. If, in fact, people select plants based on their chemical makeup (as hypothesized above), then it would be reasonable to suppose that the most important plants for these people would be weeds. In the same way, if disturbed areas and secondary vegetation are the most important sources of medicinal plants, then it would also be reasonable to assume that those plants would be important and highly sought after locally.

While the first idea (a chemical focus) brings with it the notion of the selection of resources by their users, the idea of acculturation implies passivity in relation to the physical and cultural environment. I tend to believe in a dynamic relationship between people and plants, a process that involves choices and criteria for those choices [see [6]], even while recognizing the influence of acculturative processes and the influences that one culture may have on another [see [7]]. It is in that sense that many researchers have noted a predominance of exotic plants during ethnobotanical surveys, and justify the results in terms of acculturative processes. Nonetheless, Bennett and Prance [8] defend the idea that exotic plants are very important throughout the world, and that their introduction and ample diffusion within many diverse human groups seems to be the result of their initial introduction as edible or ornamental plants. Thus, in contrast to the arguments in favor of acculturation and erosion as relatively passive processes, I propose a hypothesis based on diversification. This hypothesis is based on the premise that the inclusion of exotic plants is a strategy within many cultures for diversifying the local pharmacological stock.

Another question arises concerning the difference between knowledge of the medicinal potential of a given plant and its actual use. Many ethnobotanical surveys have furnished lists of medicinal plants (often using quantitative techniques to determine which plants are most important or most noted within a given culture) [for example: [9-11]], although rarely has a distinction been made between what is considered useful and what is actually used. It is not unusual for these quantitative techniques to reflect knowledge of utilitarian potential rather than real use [5]. Reyes-Garcia et al. [12] stressed the idea that the variables of knowledge and use are not always positively correlated. These authors found, for example, that there was no more correlation in these values among people that lived in isolated villages than there was among people who depended less on forest resources. Additionally, Ladio and Lozada [13] reported that the Mapuche (Patagonia, Argentina) know of more edible plants that they actually consume. The data of Reyes-Garcia et al. [12] thus go against the general view that these variables are closely related, and indicate that other variables may modify this relationship. Interestingly, these interpretations have in common the view that the discrepancies between knowledge and use indicate that local knowledge is eroding [13]. But, on the other hand, the differences observed between knowledge and use may point to diversification rather than erosion of knowledge.

The present work is intended to test some of the ideas put forward here by examining a rural area in the Caatinga region of NE Brazil, as well as to discuss some of the most common interpretations related to the use of medicinal plants in a given culture. Xerophytic Caatinga vegetation occupies an area of approximately 1 million km^2 ^in northeastern Brazil [14] and has suffered intense anthropogenic action. Although a complete botanical survey has never been conducted, at least 339 woody species are known from the Caatinga [15]. Native tree species are harvested for many different purposes, especially as medicinal plants and firewood. Dry forests such as the Caatinga have structural and floristic adaptations associated with the high evapo-transpiration potential and low rainfall of the semi-arid climate [15]. Thus, during many months, the people that live in this region center their attention on perennial woody species rather that on plants with shorter life cycles [16,17].

As such, the aim of the present study was to survey the plant species used by local populations in a seasonal dry forest (Caatinga) in northeastern Brazil. Evidence is presented that use-preference is an important variable in determining the active local pharmacopoeia, and that the establishment of a local medicinal plant formulary in the Caatinga is highly influenced by its seasonality. I will address the following questions: 1) are areas of disturbed or secondary vegetation the most important source of medicinal resources? The literature available [3,18,19] reports a great richness of medicinal plants in anthropogenic zones; however, there is no mention on whether these resources are used by the people who cite them. I would expect to find that a greater species richness, but not people preference); 2) is there a difference between the variety of species known and those actually used for medicinal purposes? 3) are there differences in the proportions of native and exotic species used? If these differences exist, in detriment to the native species, does this indicate erosion of knowledge? A wide variety of exotic species are expected to be found in any given region, but does the presence of these plants indicate a diversification of the medicinal plant formulary?; 4) is there redundancy in the local medicinal plant formulary, such that there is a group of plants with the same therapeutic functions (analogous functions)?

### Study site

The present study was carried out in the municipality of Alagoinha (08° 31' 00" S and 36° 48' 00" W), located in a sub-zone of the *agreste *region in the state of Pernambuco, Brazil. According to Köppen's climatic classification, the climate type is BSHs' (semi-arid of low latitudes). The average annual temperature is 25°C and the total average annual rainfall of 599 mm is irregularly distributed throughout the year. The natural vegetation is dry tropical forest (arboreal hyperxerophilic *Caatinga*) that is characterized by a predominance of xerophyte and deciduous species. The word Caatinga is of Tupi (a native South American language) origin and means "white forest" or "clear forest", due to an almost complete absence of leaves during the summer and a high level of luminosity in the forest interior. This type of forest is dry, green only during rainy periods, with spiny shrubs, yearly rainfall between 500 and 800 mm, and periods of drought that can last up to 10 months [14,15].

The rural community of Alagoinha comprises about 5,793 of the 12,522 inhabitants of the municipality [20]. The local economy in the study area is based principally on agriculture (beans, cassava, corn, tomatoes, and guava), husbandry (cattle and/or goats), and commerce. Surpluses are sold at local markets, or in neighboring towns. Large areas of vegetation have been converted to pasture or cut for timber or charcoal. The smaller properties in the rural zone are physically and structurally very similar to each other, and the great majority of them cultivate *Opuntia *spp. for use as fodder. Many people in the rural communities work as employees on large properties, and maintain a system of natural resources management characterized basically by direct exploitation, especially of native fruit trees. A more detailed description of the local culture and environment can be found in Albuquerque and Andrade [16,17] and Albuquerque et al. [19,21].

Northeastern Brazil is home to approximately 45% of the entire Brazilian rural population [22]. This intense vocation for agriculture is very sensitive to the processes of natural resources deterioration that accompany the expansion of sugar cane monoculture and cattle breeding, and which include: deforestation, erosion of soil, and the expanse of artificial ecosystems. These processes become more evident in the semi-arid areas due to its dense occupation and agriculture needs. Few ethnobotanical studies on have been carried out in this region. Recent approaches have sought to test hypotheses and have employed quantitative tools [4,19,23,24]. The community studied in the present work has previously yielded important information on practices related to the use and management of local plant resources [19,21], as well as general data on the medicinal species themselves and their relative importance [16,17]. The most important local medicinal plants are trees (such as *Schinopsis brasiliensis*, *Myracrodruon urundeuva*, and *Anadenanthera colubrina *var. *cebil*) that are also important components of the Caatinga vegetation [16,17,19]. Native populations of these plants have suffered from this human use (intensive harvesting of the bark and wood) for medicinal or construction purposes [25]. However, the homegardens in the region contribute to the conservation of local diversity, as a result of the multiple services they provide and their expressive content of native plants [21].

## Methods

### Data collection

Ethnobotanical information was collected between the years 1998 and 2001, using the community survey approach or other variants of this method [19,26]. The community was composed of approximately 31 residences at the time of the study [21], and all the family heads (one per residence) participated in the research voluntarily.

Data collection involved three basic stages. In the first step, informants from the study area were interviewed in their homes employing a semi-structured interview technique [27,28] in order to obtain information about the medicinal plant resources available to the community and factors that might explain preferential collection sites. All of the community's homes were visited in this phase, and the adult responsible for the house was included in the sample. Informants were asked about all medicinal plants used and their personal preferences in treating specific illnesses. Preference here refers to "the conscious choice in using a given resource in detriment of another that is simultaneously offered" [19]. Additionally, I sought to eliminate idiosyncratic effects and be able to distinguish between plants were merely known and those that were actually employed. This was accomplished by considering only those plants that were currently used by at least two people in the community. All of the interviewees normally used medicinal plants for different purposes. Most of these people had only limited formal education, but had lived for at least 5 years in the region.

The plants cited were collected from residences or adjacent areas with the aid of each informant. In the second stage, representative samples of the collected plants were shown individually to two principal informants [28] who had been identified as very knowledgeable on these subjects during interviews with 31 other local people (13 women and 18 men) between 25 and 70 years old. These two informants were requested to identify the plants collected and describe their usage with the aim of gathering more in depth information. The age of these informants varied from 40 to 56 years of age; one was a male and one was a female. For each of the plants cited in all of the interviews, the following information was collected: local name, use, life form, habitat, biogeografic origin, and local status (native or cultivated). The third stage of the research involved the field identification of plants in a disturbed area (cleared 45 years earlier and was presently dominated by herbs and bushes) and in an apparently well preserved area of native vegetation dominated by shrubs and trees [see details in [19] for the sample size and sampling design]. The principal informants accompanied the researchers in these two areas, aiding in the collection of plants cited during the formal data collection stages and often adding new information.

As these principal informants are considered local specialists by their peers, the information provided by them on their use and personal preferences for the use of certain species were compared to the information provided by the total population of informants (n = 31). As such, a local specialist was considered "a person who the community recognizes due to their extensive use and knowledge of native and/or exotic medicinal plants in the treatment of local illnesses" [10,29]. All of the informants agreed to participate voluntarily in the study, and were kept anonymous.

The plants collected were identified by comparison with specimens at the UFP (Departamento de Botânica, Centro de Ciências Biológicas, Universidade Federal de Pernambuco) and the IPA (Dárdano de Andrade-Lima) herbaria, and through consultation with specialists. The collections were deposited at the UFP herbarium.

### Data analysis

The chi-square test was utilized to identify differences in the patterns of knowledge and the use of resources in the community in order to examine the hypothesis that two main factors are important in determining the local pharmacopoeia: the source of medicinal plant and its life form. To determine if certain use-categories were significantly populated by the same preferred species, Spearman rank correlation coefficients [30] were used to test correlation in species ranking among the different categories. The relative importance of each mentioned species was calculated based on Friedman et al. [31] [see also [32]]. As the data did not demonstrate a normal distribution (Kolmogorov-Smirnov test), the non-parametric test of Kruskal-Wallis [33] was employed to identify which variables better indicated the relative importance of a species: herbs, non-herbs (shrubs and trees), native forest (Caatinga vegetation), or disturbed areas (yards, road sides, abandoned fields, disturbed vegetation). To compare the proportions of native/exotic species, as well as the plants known/plants effectively used, the chi-square and Williams G test were employed [30]. The frequencies of citations for the origins and the status of the resources (native or exotic) were compared using the Mann-Whitney binomial test. The frequency per species (the proportion of plants cited and utilized in relation to the total number of interviewees) was estimated according to Estomba et al. [34]. All of the analyses were carried out using the BioEstat 2.0 software [35].

## Results

### Diversity and sources of medicinal plant resources

A total of 48 plants with medicinal uses documented in this study are listed in table 1. The Caatinga appears to be a significantly more important source of medicinal plants for the studied community (chi-square test, P < 0.05) in terms of the proportion of plants from that vegetation type that are considered useful and preferred for use (15 preferred plants out of 25 used in native forests, against 3 preferred plants out of 23 used in the anthropogenic zones). Clearly, local people prefer the native vegetation for collecting medicinal plants. There do not appear to be significant differences between herbs and other plant life forms in terms of preferred and less-preferred species (chi-square test, P > 0.05) in spite of the fact that the number of non-herbs used (31 species, with 20 being preferred) is significantly larger than the number of herbs (17 species, with 3 being preferred). The Spearman rank test did not demonstrate a significant correlation between preferred species and herbs, non-herbs, native forests, or anthropogenic zones. However, according to the Kruskal-Wallis test (P < 0.01), the most important species are concentrated in two categories: non-herbs and native forests (Table 2).

**Table 1:** List of medicinal plants used in a rural community in the municipality of Alagoinha, Pernambuco, NE Brazil. Status: C = cultivated, W = wild, E = exotic, N = native

**Botanical taxon (and voucher specimen code)**	**Botanical family**	**name(s) recorded**	**Status**	**Importance**	**Part(s) used**	**Preparation**	**Administration**	**Claimed medicinal use**
*Anacardium occidentale *L. (23615)	Anacardiaceae	Caju	C (E)	0.80	Bark of the stem	Decoction or tincture	External use	Anti-inflammation and bruise
*Myracroduon urundeuva *(Engl.) Fr. All. (23382)	Anacardiaceae	Aroeira	W(N)	0.70	Bark of the stem	Decoction or tincture	External use and drunk	Anti-inflammation, bruise and gastritis
*Schinopsis brasiliensis *Engl. (23746)	Anacardiaceae	Braúna	W(N)	0.30	Bark of the stem	Infusion (tea) and syrup	Drunk	Cough and flu
*Acanthospermum hispidum *DC. (24327)	Asteraceae	Espinho-de-cigano	W(E)	0.05	Root	syrup	Drunk	Cough and asthma
*Egletes viscosa *Less. (23450)	Asteraceae	Macela	W(E)	0.10	Leaves	Infusion (tea)	Drunk	Digestive and dysentery
*Cereus jamacaru *DC. (n.c)	Cactaceae	Mandacaru	W(N)	0.10	Stem	Scrape of the bark in the water	Drunk	Problem of the kidney
*Bauhinia cheilantha *(Bong.) Steud. (24284)	Caesalpiniaceae	Mororó	W(N)	0.25	Bark of the stem; leaves and seeds	Syrup or Infusion (leaf)	Drunk	Headache, cough, diabetes and to expel catarrh
*Caesalpinia ferrea *Mart. (23639)	Caesalpiniaceae	Jucá	W(N)	0.20	Bark of the stem	Decoction (tea)	Drunk	Problem of the kidney and labirintitis
*Piptadenia stipulacea *Ducke (23717)	Mimosaceae	Carcará (rasga-beiço)	W(N)	0.30	Bark of the stem	Decoction or tincture	External use	Anti-inflammation
*Eucalyptus *sp. (23631)	Myrtaceae	Eucalipto	C(E)	0.25	Leaves	Infusion (tea)	Drunk	Fever
*Psidium guajava *L. (23613)	Myrtaceae	Goiaba	C(E)	0.50	Leaves	Infusion (tea)	Drunk	Dysentery
*Boerhavia diffusa *L. (24239)	Nyctaginaceae	Pega Pinto	W(E)	0.10	Root	syrup	Drunk	Anti-inflammation and cough
*Passiflora foetida *L. (24313)	Passifloraceae	Maracuja-de-estalo	W(N)	0.10	All Plant	Decoction with the addition of salt (syrup)	Drunk	Cough
*Cymbopogon citratus *(DC) Stapf. (n.c)	Poaceae	Capim-santo	C(E)	0.40	Leaves	Infusion (tea)	Drunk	Digestive, dysentery, fever and headache
*Ziziphus joazeiro *Mart. (23376)	Rhamnaceae	Juá	W(N)	0.35	Bark of the stem	Scrape of the bark in the water and syrup	Drunk	Cicatrizing and cough
*Citrus aurantium *L. (23742)	Rutaceae	Laranja	C(E)	0.05	Leaves	Infusion (tea)	Drunk	Fever
*Dioclea grandiflora *Mart. Ex Benth. (24235)	Fabaceae	Mucuña	W(N)	0.10	Fruit	?	?	?
*Eryhtrina velutina *Willd. (23612)	Fabaceae	Mulungu	W(N)	0.20	Bark of the stem	Infusion (tea)	Drunk	Anti-inflammation and tranquillizer
*Ocimum campechianum *Mill. (24761)	Lamiaceae	Mangericão	C(E)	0.05	Leaves	Infusion (tea)	Drunk	Digestive and dysentery
*Plectranthus *sp. (24957)	Lamiaceae	Hortelã	C(E)	0.40	Leaves	Infusion (syrup)	Drunk	Cough
*Rosmarinus officinalis *L. (n.c)	Lamiaceae	Alecrim	C(E)	0.05	Leaves	Infusion (tea)	Drunk	Fever
*Gossypium herbaceum *L. (23647)	Malvaceae	Algodão	C(E)	0.05	Leaves	Cataplasm	External use	Burn
*Anadenanthera colubrina *(Vell.) Brenan var. *cebil *(Griseb.) Altschul (23634)	Mimosaceae	Angico-de-caroço	W(N)	0.65	Bark of the stem	Infusion (syrup)	Drunk	Cough
*Mimosa tenuiflora *(Willd.) Poir. (23745)	Mimosaceae	Jurema-preta	W(N)	0.40	Bark of the stem and leaves	Infusion	External use	Anti-inflammation and tooth pain
*Momordica charantia *L. (n.c)	Cucurbitaceae	Melão-de-São-Caetano	W(E)	0.05	All Plant	Infusion	Bath	Allergy
*Cnidoscolus urens *(L.) Arthur (23442)	Euphorbiaceae	Urtiga	W(N)	0.10	Root	Decoction (tea)	Drunk	Anti-inflammation
*Croton argyrophylloides *Muell. Arg. (24310)	Euphorbiaceae	Sacatinga (Marmeleiro branco)	W(N)	0.35	Bark of the stem	Scrape of the bark in the water	Drunk	Dysentery
*Croton rhamnifolius *Willd. (23447)	Euphorbiaceae	Velame	W(N)	0.15	Leaves	Infusion (tea)	Drunk	Depurative
*Jatropha curcas *L.	Euphorbiaceae	Pinhão-bravo	W(N)	0.10	Latex	Direct or diluted in water	External use or drunk	Snake poison
*Jatropha mollissima *(Pohl) Baill. (23377)	Euphorbiaceae	Pinhão-manso	W(N)	0.30	Latex	Direct diluted in water	External use or drunk	Snake poison
*Phyllanthus niruri *L. (n.c)	Euphorbiaceae	Quebra-pedra	W(E)	0.05	Root	Infusion (tea)	Drunk	kidney diseases (specially stones)
*Amburana cearensis *(Allemão) A. C. Sm. (23737)	Fabaceae	Imburana-de-cheiro	W(N)	0.80	Bark of the stem	Infusion (tea) and syrup	Drunk	Cough and flu
*Caesalpinia pyramidalis *Tul. (23650)	Caesalpiniaceae	Catingueira	W(N)	0.35	Bark of the stem	syrup	Drunk	Cough
*Hymenaea courbaril *L. (24306)	Caesalpiniaceae	Jatobá	W(N)	0.60	Bark of the stem	syrup	Drunk	Cough, bronchitis, weakness and debility
*Senna martiana *(Benth.) H. S. Irwin & Barnebv (23638)	Caesalpiniaceae	Canafístula	W(N)	0.10	Bark of the stem	syrup	Drunk	Cough
*Capparis flexuosa *L. (23380)	Capparaceae	Feijão-de-boi	W(N)	0.20	Bark of the stem	Scrape of the bark in the water	Drunk	Snake poison
*Cleome spinosa *Jacq. (23448)	Capparaceae	Mussambê	W(E)	0.05	Leaves and flower	Tincture or syrup	External use or drunk	Cough
*Maytenus rigida *Mart. (23383)	Celastraceae	Bom-nome	W(N)	0.15	Bark of the stem	syrup and bark in the water	Drunk	Cough and rheumatic
*Chenopodium ambrosioides *L. (n.c)	Chenopodiaceae	Mastruz	C(E)	0.15	Leaves	Infusion (syrup)	External use	Cough
*Kalanchae brasiliensis *Cam. (23440)	Crassulaceae	Pratudo	C(E)	0.05	Leaves	Warm up leaves	External use	Pain in general
*Ruta graveolens *L. (n.c)	Rutaceae	Arruda	C(E)	0.30	Leaves	Tincture	?	Headache
*Sapindus saponaria *L. (23729)	Sapindaceae	Sabonete	W(N)	0.15	Bark of the stem	Decoction	Wash the hair	fungi
*Serjania comata *Radlk. (23443)	Sapindaceae	Ariu	W(N)	0.05	Root	Infusion (tea)	Drunk	Rheumatism
*Sideroxylon obtusifolium *(Roem & Schult.) T. D. Penn. (n.c)	Sapotaceae	Quixaba	W(N)	0.80	Bark of the stem	Decoction or tincture	External use	Cicatrizing, bruise and anti-inflammation
*Solanum paniculatum *L. (23449)	Solanaceae	Jurubeba	W(E)	0.05	Root and fruits	Infusion (tea)	Drunk	Anti-inflammation and liver diseases
*Lippia alba *(Mill.) N.E. Br. (24233)	Verbenaceae	Erva-cidreira	W(E)	0.65	Leaves	Infusion (tea)	Drunk	Digestive, dysentery, headache, fever and blood pressure
*Lippia sp*. (24303)	Verbenaceae	Alecrim	W(N)	0.10	Leaves	Infusion (tea)	Drunk	Digestive and dysentery
*Hybanthus cf. ipecacuanha *(L.) Baill. (23441)	Violaceae	Pepaconha	W(E)	0.15	Root	syrup	Drunk	Cough

**Table 2:** Descriptive statistics and Kruskal-Wallis test based on the relative importance of each species by studied categories (H = 29.58; P < 0.01).

	Herbs^1^	Non-herbs^2^	Native Forests^3^	Anthropogenic Zones^4^
Mean	0.1367	0.3567	0.3533	0.0767
Standard deviation	0.1260	0.2478	0.2264	0.0372
Variance	0.0159	0.0614	0.0512	0.0014
Coefficient of variance	92.21%	69.46%	64.06%	48.47%

1 and 2, 1 and 3, 2 and 4, 3 and 4 = P < 0.01; 1 and 4, 2 and 3 = P > 0.05

### Native and exotic flora

No significant differences were found between the proportion of species of native vs. cultivated plants that were known and used by the local community examined (chi-square test P > 0.05). This means that the community knows and uses native and exotic species in the same proportion. Thirty-two wild species were known, and 22 actually used; while 12 cultivated species were known, and 7 used. Similarly, no significant differences were found between the proportion of species of native vs. exotic plants that were known and used by the local community examined (chi-square test P > 0.05), with 26 wild species known, and 20 actually used; while approximately 20 cultivated species were known, and 7 used. Comparisons of the frequency of the interviewees citing a given species, revealed that the local community knows and uses significantly more plants from areas of native vegetation than from altered areas, more wild than cultivated species, and more natives than exotics (Mann-Whitney test, P < 0.05).

### Local uses

The community members indicated 29 different therapeutic uses for the plants cited, of which 16 uses were attended by only a single species of medicinal plant (6 of these being exotic plants) (Table 3). The proportions between exclusive and non-exclusive species used for treating a specific disease were similar among native and exotic plants (Willam's G test, P > 0.05). However, three diseases are basically treated only by exotic plants: digestive problems (9 species), headaches (3), and fevers (5) (Willam's G test, P < 0.01). The medical uses with the greatest number of species employed were: coughs (14 species), digestive disorders (11), and anti-inflammatory treatments (9). Native species were more frequently cited to treat wounds and inflammations (binomial test, P < 0.05). Exotic plants were more frequently cited to treat digestive problems (binomial test, P < 0.05). In spite of a diverse formulary of useful species, the local community preferred basically four species (all native) to treat their principal health problems (even those diseases that could be treated by more than one species): *Myracrodruon urundeuva*, *Anacardium occidentale*, *Sideroxylon obstusifolium *and *Amburana cearensis*.

**Table 3:** Species richness and therapeutical indications of medicinal plants used by an rural community in Caatinga vegetation, Pernambuco (NE Brazil). *the preferred species was not cited for the indication.

**Indications**	**Number of all species – (only Exotic plants)**	**Preferred species**
Cough	14 (4)	*Amburana cearensis*, *Schinopsis brasiliensis*, *Hymenaea courbaril*.
Anti-inflammation	9 (2)	*Myracrodruon urundeuva*, *Anacardium occidentale*, *Sideroxylon obstusifolium*.
Dysentery	7 (5)	*Psidium guajava*.
Bruise	5	*Myracrodruon urundeuva*, *Anacardium occidentale*, *Sideroxylon obstusifolium*.
Fever	5 (5)	*Eucalyptus *sp.
Digestive	4 (4)	*Cymbopogon citratus*.
Problems of kidney	4 (1)	*Cereus jamacaru*, *Phyllanthus niruri*.
Headache	4 (3)	*Lippia alba*.
Snake poison	3	*Jatropha mollissima*.
Flu	2	*Amburana cearensis*.
Cicatrizing	2	*Sideroxylon obstusifolium*.
Rheumatism	2	*Maytenus rigida*.
Gastritis	1	*
Asthma	1 (1)	*
Diabetes	1	*
Expel catarrh	1	*
Labirintitis	1	*
Tranquilizer	1	*
Burn	1 (1)	*
Tooth pain	1	*
Allergy	1 (1)	*
Depurative	1	*
Bronchitis	1	*
Weakness	1	*
Debility	1	*
Fungi	1	*
Pain in general	1 (1)	*
Liver diseases	1 (1)	*
Blood pressure	1 (1)	*

## Discussion

The majority of our hypotheses were not confirmed, as altered areas do not appear to be the greatest source of medicinal plants, weeds are not largely present in the local medicinal formulary either in terms of richness nor preference by the population, and the proportion of species known vs. used is similar between native plants and exotic plants, and wild and cultivated. Nonetheless, based on the preference and relative importance data, native plants appear to be the most valuable resource for the local population in the studied area, especially the woody species; areas of natural vegetation are clearly the preferred collection sites in these cases, and are the most cited and best known by the people there. It was observed that people generally neglected medicinal plants growing around their homes or on the roadside, and have a strong preference for, and use, the natural vegetation and the natural woody species over plants from disturbed areas or weeds. These results contrast with the tendencies demonstrated by other ethnobotanical studies over the last 10 years. The tendency has been to believe that the native vegetation was the most important source of medicinal resources for the local human populations in the world was being slowly overtaken by comparative studies between primary and secondary vegetations [18] and by several surveys that pointed to the supremacy of weeds and altered areas in traditional medicine formularies [1,2]. Voeks [3] argued incisively that the folk medicine repertoires in tropical forests were based on a "disturbance regime" after reviewing different surveys that indicated the overwhelming importance of medicinal plants in managed habitats. This author concluded that people paid more attention to these habitats due to their greater familiarity, accessibility, and the rich presence of biologically active secondary compounds in the plants growing there. In our study, however, in spite of their accessibility, these disturbed area resources are not as important to the local population, suggesting that medicinal plant use customs may vary in tropical regions [19].

Numerous publications have supported these ideas of Voeks [3] with field data [36-38] without, however, closely detailing the context of the use of these resources or distinguishing between what is cited as useful and what is effectively consumed. Results and use patterns similar to ours were reported by Estomba et al. [34] for the community of Mapuche in Patagonia, Argentina. These authors documented that the local population would invest must effort in collecting native species, although their motives were not entirely clear. This emphasis on the collection of native species likewise occurred in the community examined here, even though these people were aware of cultivated substitutes, or plants that were easily available at the roadside or in the neighbor's yards. This reinforces the view of Estomba et al. [34] that human decision processes are complex and cannot be easily reduced to rational economic payoffs. Social and cultural factors may influence the processes involved in choosing and harvesting medicinal plants, although the habitats themselves may help to explain some of the patterns observed. I believe, for example, that the preference for non-herbaceous plants may be explained by the seasonality of the Caatinga vegetation. The most important species in the Caatinga are native plants whose bark (available all year round) is principally sought. The short period of rainfall in the region determines that short lifecycle plants whose leaves are used would only be available for very limited periods of time [16,17]. Interestingly, even when these plants are most available they are not greatly harvested. Additional research has reinforced our hypothesis that the seasonality of resource availability can explain many of the patterns of resource utilization in arid and semi-arid regions [see [4,34]]. This seasonality hypothesis predicts that the preferred and most used resources will be native plants that are always available, although this relationship could be modified by other economic or social variables. For example, the use of cultivated medicinal plants is rarer in the dryer regions of the Caatinga due to the irregularity of the rainfall and the necessity of economizing water for other more important uses.

The fact that the local population also knows of more medicinal plants than it actually uses can also be explained in part, by the observations above. The favored explanation for this difference between knowledge and actual use has been the "erosion" of local knowledge fueled by the substitution of plants by commercially available alternatives [12]. Although it has been documented that these relationships may vary [12,13] due to social, economic and cultural variables, the erosion hypothesis (while very plausible) cannot explain all the phenomena observed.

It would seem that the absence of this correlation between knowledge and effective use in the community examined is related to the view of the non-preferred species as an option, to be used only when necessary. In this sense, interpreting these results as knowledge erosion would not fit here. Alternatively, inside the idea of diversification, I propose two knew concepts. I can designate this diversity of known plants as "mass knowledge". By mass knowledge I refer to the total group of plants known as useful in a given culture, including those that are not used correctly. In certain circumstances (social, economical, historical, or ecological contexts), some of these plants can be used as what I call "stock knowledge". In this last case the plants that were only part of the community's theoretical repertoire become part of the practical dimension [for a definition of theoretical and practical knowledge or dimension see [39]]. As such, these discrepancies between knowledge and use do not in themselves characterize erosion of knowledge. This erosion could occur, however, if the processes of transmission of knowledge were to break down. For example, *Dioclea grandiflora *is a commonly known species in the region, but its medicinal use is little known within the community. Additionally, it is known to be edible (in times of great difficulty), but knowledge of how to prepare it for consumption is restricted to only two local people. As such, I share the opinion of Reyes-Garcia et al. [12] that an examination of knowledge and actual use will aid our understanding of the processes of erosion. *D. grandiflora *illustrates well the case of a "famine food" or "emergency food", poorly studied in ethnobotanical literature [40]. Minnis [40] suggests that these little preferred plants in a given culture – either by biological or cultural aspects – are in risk of not being transmitted from one generation to the other due to the interruption of learning processes.

In the present work, 58.6% of the therapeutic categories where treated by only a single species. As such, the redundancy of useful species observed is low in comparison to most other surveys [10,11,41,42] although some human groups demonstrate an even greater specificity [see [43]]. This is the case, for example, of the Krahô Indians in Brazil, who usually employ only a single plant to treat a specific problem [44]. This idea of species redundancy in traditional medicinal systems prompted us to propose the utilitarian redundancy hypothesis as a model to examine resilience and the impact of redundancy on the conservation of native plants [see [41] for a more detailed discussion of this proposal).

Native plants are the basis of the traditional medicinal practices of the community studied, although exotic plants compose 48% of their total medicinal plant formulary. This fact could be cited as an argument supporting the idea of erosion of traditional knowledge. Although native plants are preferred, exotic plants are still very important locally as they are specifically indicated to treat many infirmities. This collaborates with our hypothesis of diversification, which considers that the presence of these species in the local traditional medicine formulary takes the role of amplifying the spectrum of alternatives for treating specific illnesses (although some are even specific and singular treatments). In this sense, I feel that the principal reason for the inclusion of these plants is the possibility of reinforcing the local medicinal plant formulary with species containing highly bioactive secondary metabolic compounds to be used in well defined treatments. Nevertheless, this needs to be tested formally. These plants may also represent analogous treatments that are more "palatable" than those offered by the native species [see [34]].

As such, exotic plants that are often ignored in ethnobotanical studies deserve more attention [3,8], for they may represent a floristic group rich in pharmacologically active substances [3], at the same time as their role in different cultures must be carefully analyzed in order to avoid undue conclusions concerning acculturation and erosion of knowledge. The preservation of knowledge concerning both native and exotic plants is important in order to maintain the resiliency of the knowledge system of the community studied here.

## Declaration of competing interests

The author(s) declare that they have no competing interests.

## Authors' contributions

This is a sole authored paper
